# Molecular evidence for historical presence of knock-down resistance in *Anopheles albimanus*, a key malaria vector in Latin America

**DOI:** 10.1186/1756-3305-6-268

**Published:** 2013-09-18

**Authors:** Juan C Lol, María E Castellanos, Kelly A Liebman, Audrey Lenhart, Pamela M Pennington, Norma R Padilla

**Affiliations:** 1 Centro de Estudios en Salud, Universidad del Valle de Guatemala (CES-UVG), 18 avenida 11-95 zona 15 Vista Hermosa 3, Guatemala, Guatemala; 2 Centers for Disease Control and Prevention (CDC), Center for Global Health, Division of Parasitic Disease and Malaria, Entomology Branch, 1600 Clifton Road, Atlanta, GA 30329, USA

**Keywords:** *Anopheles albimanus*, Pyrethroid resistance, Voltage-gated sodium channel gene, *kdr* mutations

## Abstract

**Background:**

*Anopheles albimanus* is a key malaria vector in the northern neotropics. Current vector control measures in the region are based on mass distributions of long-lasting insecticidal nets (LLINs) and focal indoor residual spraying (IRS) with pyrethroids. Resistance to pyrethroid insecticides can be mediated by increased esterase and/or multi-function oxidase activity and/or mutations in the voltage-gated sodium channel gene. The aim of this work was to characterize the homologous *kdr* region of the voltage-gated sodium channel gene in *An. albimanus* and to conduct a preliminary retrospective analysis of field samples collected in the 1990’s, coinciding with a time of intense pyrethroid application related to agricultural and public health insect control in the region.

**Methods:**

Degenerate primers were designed to amplify the homologous *kdr* region in a pyrethroid-susceptible laboratory strain (Sanarate) of *An. albimanus*. Subsequently, a more specific primer pair was used to amplify and sequence the region that contains the 1014 codon associated with pyrethroid resistance in other *Anopheles* spp. (L1014F, L1014S or L1014C).

**Results:**

Direct sequencing of the PCR products confirmed the presence of the susceptible *kdr* allele in the Sanarate strain (L1014) and the presence of homozygous-resistant *kdr* alleles in field-collected individuals from Mexico (L1014F), Nicaragua (L1014C) and Costa Rica (L1014C).

**Conclusions:**

For the first time, the *kdr* region in *An. albimanus* is described. Furthermore, molecular evidence suggests the presence of *kdr*-type resistance in field-collected *An. albimanus* in Mesoamerica in the 1990s. Further research is needed to conclusively determine an association between the genotypes and resistant phenotypes, and to what extent they may compromise current vector control efforts.

## Background

*Anopheles albimanus* is one of the key malaria vectors of Latin America and is widely distributed throughout the region [1,2]. In recent years, insecticide resistance has emerged in malaria vectors worldwide as a result of increased intensity of insecticide use, principally via the widespread use of indoor residual spraying (IRS) and long-lasting insecticidal nets (LLINs) in malaria endemic areas [3-5]. Malaria control in the region currently relies heavily on the use of LLINs, which are treated with pyrethroid insecticides [6]. The widespread use of insecticide treated nets (ITNs) [7-11], LLINs [12-14] and both the historical and ongoing use of DDT and pyrethroid insecticides for IRS [13,15-17] elicit selection pressures on local vector populations. As such, the routine surveillance of insecticide resistance must be implemented in the context of vector control programs to verify that control tools are maintaining their efficacy. The timely detection of insecticide resistance and the characterization of the mechanisms underlying insecticide resistance in a vector population can provide valuable data regarding which insecticides should be used to maintain maximum vector control impact.

Resistance to pyrethroid insecticides in malaria vectors can be primarily mediated by either metabolic mechanisms or target site insensitivity, such as mutations on the voltage-gated sodium channel (*VGSC*) gene [3,18]. Despite reports of pyrethroid resistance throughout the region, none of these mechanisms have been well-described at the molecular level for malaria vectors in Latin America [19]. Previous studies using biochemical assays and bioassays with synergists on pyrethroid resistant *An. albimanus* from Guatemala and Mexico suggest that an increase in the activity levels of esterases and multi-function oxidases are at least partially responsible for the resistance detected in these populations [20-24]. Elevated oxidase activity has been associated with cross-resistance to pyrethroids and DDT in *An. albimanus*[23]. One previous study carried out on *An. albimanus* from Mexico suggested that a target-site mechanism may be involved in cross-resistance between pyrethroids and DDT [25]. Knock-down resistance (*kdr*) is a target-site mechanism reported in other anopheline species that results in cross-resistance to both pyrethroids and DDT [26,27]. In anophelines, *kdr* is linked to single nucleotide polymorphisms on transmembrane segment 6 of domain II of the *VGSC* gene. The mutations previously reported for anophelines occur on codon 1014, resulting in an amino acid change of leucine to phenylalanine, serine or cysteine [28-34]. To date, similar mutations have not been described in *An. albimanus*.

The present study describes for the first time the homologous *kdr* region of the *VGSC* gene in *An. albimanus* where mutations in other anopheline species have been detected that are associated with *kdr*-type resistance. Further, we report molecular evidence of *kdr* resistant-type alleles in field mosquitoes collected in Mexico, Nicaragua and Costa Rica in the 1990s.

## Methods

### Primer design

**Figure 1:**
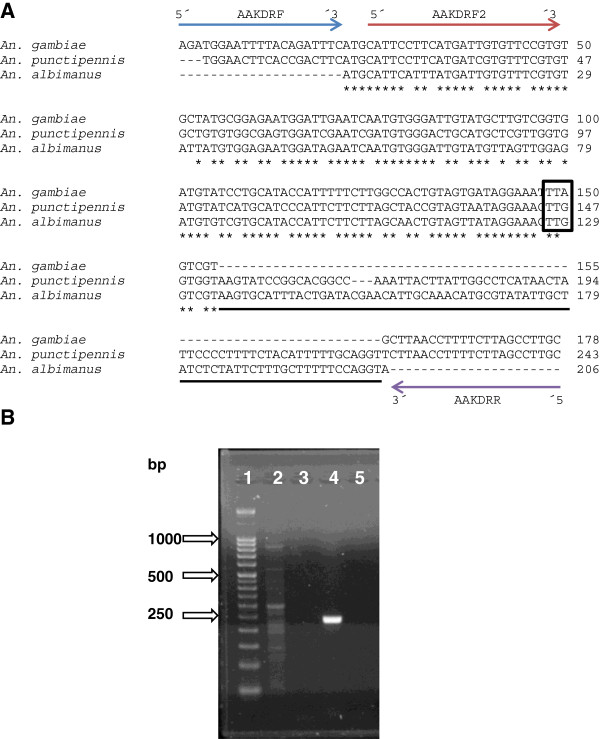
**Strategy to amplify segment 6 of domain II of the *VGSC *gene in *Anopheles albimanus*. (A)** Diagrammatic representation of the design of degenerate and specific primers for *An. albimanus* [GenBank: KF137581] based on *An. gambiae* [GenBank: Y13592] and *An. punctipennis* [GenBank: AY283041]. The identical positions are indicated by an asterisk and mutation site is enclosed by a box. Intron position is indicated by a black line below the sequence. AAKDRF (5′-AGATGGAAYTTYACNGAYTTC-′3); AAKDRF2 (5′-CATTCATTTATGATTGTGTTTCGTG-′3); AAKDRR (5′-GCAANGCTAAGAANAGRTTNAG-′3). **(B)** PCR products using degenerate and specific primers. The PCR products were separated on a 2% agarose gel containing ethidium bromide. Lane 1: 50 bp DNA ladder (Novagen); Lane 2: degenerate PCR products (using AAKDRF and AAKDRR primers); Lane 3: negative control of degenerate PCR (H_2_O); Lane 4: specific PCR product (using AAKDRF2 and AAKDRR primers); Lane 5: negative control of specific PCR (H_2_O).

DNA and cDNA sequences of the *VGSC* gene of different *Anopheles* spp. were retrieved from GenBank (Table 1). Conserved regions were identified from a multiple alignment (MEGA 5.0 [35]) and degenerate primers were designed based on conserved codons using *An. punctipennis* as a basis [GenBank: AY283039-AY283041]. The strategy used to design the primers to amplify the *VGSC* gene in *An. albimanus* is presented in Figure 1A.

**Table 1:** **DNA sequences of the *VGSC *gene from different *Anopheles *spp. used in the primer design**

**Specie (subgenus)**	**Sequence identification**
*Anopheles aconitus* (*Cellia*)	GenBank: EU155388
*An. annularis* (*Cellia*)	GenBank: DQ026443
*An. arabiensis* (*Cellia*)	GenBank: DQ263749
*An. culicifacies* (*Cellia*)	GenBank: GQ279245
	GenBank: GQ279246
	GenBank: GQ279247
*An. dirus* (*Cellia*)	GenBank: DQ026439
	GenBank: DQ026440
	GenBank: DQ026441
	GenBank: DQ026442
*An. epiroticus* (*Cellia*)	GenBank: EU155384
*An. funestus* (*Cellia*)	GenBank: DQ399296
	GenBank: DQ399298
*An. gambiae* (*Cellia*)	GenBank: Y13592
	GenBank: DQ263748
*An. harrisoni* (*Cellia*)	GenBank: EU155387
*An. jeyporiensis* (*Cellia*)	GenBank: EU155389
*An. kochi* (*Cellia*)	GenBank: DQ026446
*An. maculatus* (*Cellia*)	GenBank: DQ026445
*An. minimus* (*Cellia*)	GenBank: GU064930
	GenBank: EU155386
*An. paraliae* (*Anopheles*)	GenBank: GQ225104
*An. peditaeniatus* (*Anopheles*)	GenBank: GQ225106
*An. punctipennis* (*Anopheles*)	GenBank: AY283041
	GenBank: AY283039
	GenBank: AY283040
*An. sinensis* (*Anopheles*)	GenBank: JN002364
	GenBank: GQ225102
*An. stephensi* (*Cellia*)	GenBank: JF304953
*An. subpictus* (*Cellia*)	GenBank: EU155385
	GenBank: DQ333331
*An. tessellatus* (*Cellia*)	GenBank: DQ075250
*An. vagus* (*Cellia*)	GenBank: GQ225100

### Mosquito population

The *An. albimanus* Sanarate laboratory strain, maintained in the insectary of Center for Health Studies (CHS) of Universidad del Valle de Guatemala (Guatemala, Guatemala) was used to validate the designed primers. The Sanarate strain is susceptible to DDT, deltamethrin, permethrin, bendiocarb and malathion (unpublished observations) according to bottle bioassay susceptibility tests [36]. Genomic DNA from individual mosquitoes was isolated following the method described by Collins *et al*. [37].

### Amplification, cloning and sequencing of the *VGSC* gene

The amplification of segment 6 of domain II of the *VGSC* gene with degenerate primers was carried out in a 50 μl reaction mixture containing 1X Colorless GoTaq® Flexi Buffer, 1.5 mM MgCl_2_, 0.2 mM dNTPs, 2.5 μM of each degenerate primer (AAKDRF and AAKDRR), 1 unit of GoTaq® HotStart Polymerase (Promega, Fitchburg, Wisconsin) and 10 to 30 ng of genomic DNA. The degenerate PCR conditions were 95°C for 3 min, followed by 35 cycles of 95°C for 45 sec, 40.5°C for 45 sec and 72°C for 1 min with a final extension step at 72°C for 5 min in a Px2 Thermal Cycler (Thermo Fisher Scientific, Waltham, Massachusetts).

Non-specific amplification was obtained in *An. albimanus* from the Sanarate strain using the degenerate primers (Figure 1B). Four different-sized PCR products were isolated for specific amplification using the band-stab PCR technique [38]. These purified PCR products were directly sequenced by Macrogen Inc. (Korea) using AAKDRF and AAKDRR as sequencing primers. BLAST analysis showed that a fragment of approximately 250 bp corresponded to the *VGSC* gene in *An. albimanus*. To confirm these findings and to obtain a high-quality DNA sequence of this fragment, PCR products were cloned using a TA Cloning® Kit (Invitrogen, Carlsbad, California) according to the manufacturer’s instructions. The plasmids of the positive clones that contained the fragment of *VGSC* gene were isolated with the PureLink™ HQ Mini Plasmid Purification Kit (Invitrogen, Carlsbad, California) according to the manufacturer’s instructions. Plasmids were sequenced with M13 universal primers using 3500XL Genetic Analyzer (Applied Biosystems, Foster City, California) with BigDye® Terminator v1.1.

### PCR assay to detect *kdr*-type resistance

A second, non-degenerate forward primer (AAKDRF2) was designed based on the sequence of the *VGSC* gene of *An. albimanus* (GenBank: KF137581) obtained with the degenerate primers (Figure 1A). The amplification with the specific forward (AAKDRF2) and AAKDRR primer was performed using the same reaction specifications as in the degenerate PCR, except that 0.5 μM of each primer were used. The PCR conditions consisted of an initial denaturation at 95°C for 3 min, followed by 40 cycles at 95°C for 45 sec, 51.5°C for 45 sec and 72°C for 1 min, with a final extension step at 72°C for 5 min in an iCycler (BioRad, Hercules, California). The PCR assay with AAKDRF2 and AAKDRR primers amplified a single band of 225 bp in *An. albimanus* from the Sanarate strain (Figure 1B), which corresponds to the *VGSC* gene of *An. albimanus*. These primers were used to amplify the *VGSC* gene in DNA samples of *An. albimanus* from Guatemala (collected in 1995), Mexico (collected in 1991), Nicaragua (collected in 1995), Costa Rica (collected in 1995), Ecuador (collected in 1991) and Colombia (collected in 1992) previously used in population genetic studies [39,40]. The PCR products were sequenced by Macrogen Inc. (Korea) using AAKDRF2 and AAKDRR primers.

## Results and discussion

**Figure 2:**
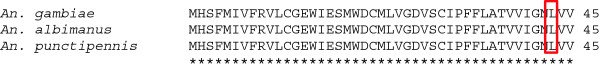
**Amino acid sequence comparison of *kdr *region of *Anopheles albimanus *with other anopheline species.** The sequence of the segment 6 of domain II of the VGSC gene of *An. albimanus* was compared to *An. gambiae* [GenBank: CAA73920] and *An. punctipennis* [GenBank: AAP60053]. Identical positions are indicated by an asterisk and mutation site (codon 1014) is enclosed by a red box. The amino acid at the mutation site corresponds to the pyrethroid and DDT susceptible (wild-type) genotypes.

Sequence analysis showed that segment 6 of domain II of the *VGSC* gene (excluding the intron sequence) of *An. albimanus* has a sequence identity of 92% with *An. gambiae* and 83% with *An. punctipennis* at the nucleotide level. Variations in the nucleotide sequence of *An. albimanus* did not produce changes in the amino acid sequence (100% identity with *An. gambiae* and *An. punctipennis*, Figure 2). The position of intron II was established through comparison with the *VGSC* cDNA sequence from *An. gambiae* [GenBank: Y13592]. The size of intron II in *An. albimanus* (71 bp) was greater than in *An. gambiae* (57 bp) and *An. punctipennis* (68 bp). Variation in the size of intron II has been detected in *An. vestitipennis* and *An. pseudopunctipennis* (unpublished observations), and may potentially be used for taxonomic identification of malaria vectors from Latin America, as proposed for other anopheline species [41].

**Figure 3:**
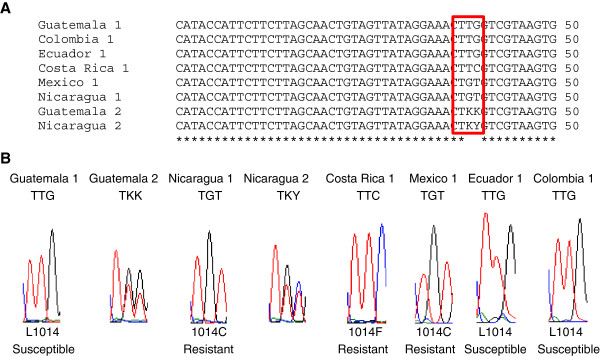
***Kdr *alleles detected on the segment 6 of domain II of the *VGSC *gene of *Anopheles albimanus. *(A)** DNA alignment of the *VGSC* gene of *An. albimanus* from different regions of Latin America. The identical positions are indicated by an asterisk and polymorphic site (codon 1014) is enclosed by a red box. **(B)** Electropherograms for *kdr* alleles detected on the *VGSC* gene of *An. albimanus*.

Sequence results from the Sanarate strain of *An. albimanus* showed that the individuals contained the susceptible/wild type *kdr* allele, TTG (L1014), previously reported in *An. sacharovi*, *An. sinensis* and other anopheline species from the Mekong region [34,42,43]. In the field-collected mosquitoes from Latin America, polymorphisms at codon 1014 were detected in several of the samples (Figure 3A). The field samples from Guatemala, Ecuador and Colombia also contained the susceptible TTG (L1014) allele. A non-synonymous homozygous mutation, TGT (cysteine, L1014C), was detected in field samples from Mexico and Nicaragua. This mutation has previously been associated with permethrin, deltamethrin and beta-cypermethrin resistance in *An. sinensis*[34,44,45]. A field sample from Costa Rica contained a homozygous TTC polymorphism (phenylalanine, L1014F), previously reported in populations of *An. gambiae* resistant to permethrin and DDT, *An. sinensis* resistant to deltamethrin and *An. peditaeniatus* resistant to DDT, permethrin, alpha-cypermethrin, lambda-cyhalothrin and etofenprox [28,43,45]. With the exception of certain individuals from Nicaragua and Guatemala, all *kdr* alleles were found to be homozygous (Figure 3B). The heterozygote alleles from Nicaragua were TKY and from Guatemala were TKK. Interestingly, the *kdr* allele reported in *An. gambiae* from East Africa (L1014S) [29] was not detected.

*An. albimanus* populations are panmictic over at least 600 km in Central America, West of Panama [46]. In this region, insecticide resistance in *An. albimanus* has been reported and the main source of its selection has been the extensive use of pesticides in large scale agricultural activities [47-50]. During the nineties, populations in the area in continued exposure to agricultural insecticides plus pressures from the use of insecticides for vector control could have maintained a constant selection pressure on Mesoamerican *An. albimanus* populations, possibly explaining the finding of three homozygous *kdr* variants in Mexico, Nicaragua and Costa Rica with mutations that have been associated with pyrethroid and DDT resistance in other anopheline species. Even though to date the role of *kdr* has not been directly implicated in the insecticide resistance documented in the region, it is highly likely that *kdr* is an important resistance mechanism in Latin American malaria vectors.

## Conclusions

Our findings describe for the first time the *kdr* region in *An. albimanus*, including the presence of polymorphisms associated with insecticide resistance in other anopheline species. We have documented the presence of homozygous *kdr* alleles associated with resistance in other anopheline species in *An. albimanus* individuals collected across Mesoamerica at a time of intense agricultural and public health insecticide use. This suggests that pyrethroid and DDT resistance in the region could have been mediated in the past by a *kdr* mechanism. Future work will endeavor to link resistant phenotypes with the *kdr* polymorphisms described here, as well as lead to the development of allele-specific diagnostic assays for *An. albimanus* and other malaria vectors across the region.

## Competing interests

The authors declared that they have no competing interests.

## Authors’ contributions

JCL carried out the molecular assays, data analysis and drafted the manuscript. MEC conducted molecular assays and contributed to the manuscript. KL conducted molecular assays and sequencing. AL assisted with the analysis and interpretation of results and contributed to the manuscript. PMP designed and guided the study, performed data analysis and contributed to the manuscript. NRP conceived the study and participated in analysis and interpretation of data and contributed to the drafting of the manuscript. All authors have read and approved the final manuscript.
